# CRISPR-Cas gene-editing reveals RsmA and RsmC act through FlhDC to repress the SdhE flavinylation factor and control motility and prodigiosin production in *Serratia*

**DOI:** 10.1099/mic.0.000283

**Published:** 2016-06

**Authors:** Hannah G. Hampton, Matthew B. McNeil, Thomas J. Paterson, Blair Ney, Neil R. Williamson, Richard A. Easingwood, Mihnea Bostina, George P. C. Salmond, Peter C. Fineran

**Affiliations:** ^1^​ Department of Microbiology and Immunology, University of Otago, PO Box 56, Dunedin 9054, New Zealand; ^2^​ Department of Biochemistry, University of Cambridge, Tennis Court Road, Cambridge CB2 1QW, UK; ^3^​ Otago Centre for Electron Microscopy, University of Otago, PO Box 56, Dunedin 9054, New Zealand

**Keywords:** prodigiosin, motility, secondary metabolism, antibiotic, CRISPR-Cas, SDH5/SdhAF2

## Abstract

SdhE is required for the flavinylation and activation of succinate dehydrogenase and fumarate reductase (FRD). In addition, SdhE is conserved in proteobacteria (α, β and γ) and eukaryotes. Although the function of this recently characterized family of proteins has been determined, almost nothing is known about how their genes are regulated. Here, the RsmA (CsrA) and RsmC (HexY) post-transcriptional and post-translational regulators have been identified and shown to repress *sdhEygfX* expression in *Serratia* sp. ATCC 39006. Conversely, the flagella master regulator complex, FlhDC, activated *sdhEygfX* transcription. To investigate the hierarchy of control, we developed a novel approach that utilized endogenous CRISPR (clustered regularly interspaced short palindromic repeats)-Cas (CRISPR associated) genome-editing by a type I-F system to generate a chromosomal point mutation in *flhC*. Mutation of *flhC* alleviated the ability of RsmC to repress *sdhEygfX* expression, whereas RsmA acted in both an FlhDC-dependent and -independent manner to inhibit *sdhEygfX*. Mutation of *rsmA *or *rsmC*, or overexpression of FlhDC, led to increased prodigiosin, biosurfactant, swimming and swarming. Consistent with the modulation of *sdhE* by motility regulators, we have demonstrated that SdhE and FRD are required for maximal flagella-dependent swimming. Together, these results demonstrate that regulators of both metabolism and motility (RsmA, RsmC and FlhDC) control the transcription of the *sdhEygfX *operon.

## INTRODUCTION

*Serratia* sp. ATCC 39006 is a member of the Enterobacteri aceae that was isolated from a salt marsh (Bycroft* et al.*, 1987), and is a model bacterium for the study of the biosynthesis and regulation of antibiotics, particularly prodigiosin (2-methyl-3-pentyl-6-methoxyprodigiosin) (Williamson* et al.*, 2006). There is pharmaceutical interest in the red tripyrrole prodiginines due to their anticancer, immunosuppressant , antimicrobial and antimalarial properties (Williamson* et al.*, 2006, 2007). We have demonstrated that an interconnected regulatory network controls the biosynthesis of prodigiosin, which responds to various cues, including bacterial cell density through quorum sensing (Fineran* et al.*, 2005b; Slater *et al.*, 2003; Thomson* et al.*, 2000), cyclic-di-GMP signalling (Fineran* et al.*, 2007; Williamson* et al.*, 2008) , phosphate availability (Gristwood et al., 2009; Slater et al., 2003), carbon source (Fineran et al., 2005a) and stationary phase (Wilf & Salmond, 2012), among others. A number of the DNA -binding transcriptional regulators of prodigiosin, including SmaIR (LuxIR-type quorum-sensing system), Rap (regulator of antibiotic and pigment) and PigP, also co-ordinately control the ability to undertake swimming and swarming motility (Fineran *et al.*, 2005b; Williamson *et al.*, 2008). The ability to swarm involves cell elongation, and requires the synthesis of flagella and biosurfactant (Jarrell & McBride, 2008; Williamson *et al.*, 2008). Interestingly, the co-ordinate production of the biosurfactant and pigment is important for *Serratia* 39006 to elicit prodigiosin-dependent antibiotic killing of competing bacteria – a potentially important physiological role for this red pigment during movement of the bacterium into new niches (Williamson *et al.*, 2008).

During our studies into the regulation of secondary metabolism in *Serratia* 39006, SdhE (formerly YgfY) was identified, due to transposon insertions within the *sdhEygfX* operon that reduced prodigiosin biosynthesis (Fineran* et al.*, 2005b; McNeil* et al.*, 2012). In Enterobacteriaceae, *sdhE* is co -transcribed with *ygfX*, which encodes a membrane protein of unknown function that interacts with SdhE (McNeil *et al.*, 2013). We demonstrated that SdhE is required for the flavin ylation and activation of the complex II enzymes succinate dehydrogenase (SDH) and fumarate reductase (FRD) – key enzymes in oxidative phosphorylation and the TCA cycle (McNeil* et al.*, 2012, 2014). Under aerobic conditions, SDH donates electrons to the electron transport chain during the oxidation of succinate to fumarate (Maklashina* et al.*, 2013). For catalysis, the SdhA subunit of SDH requires a covalently bound FAD co-factor (Blaut* et al.*, 1989; Cecchini* et al.*, 2002; Yankovskaya* et al.*, 2003). FRD catalyses the reverse reaction to SDH – the anaerobic reduction of fumarate to succinate. FRD also requires an FAD co-factor within the FrdA subunit (Blaut* et al.*, 1989; Iverson* et al.*, 1999). It was previously thought that FAD attachment was autocatalytic. However, the discovery and characterisation of SdhE demonstrated that SdhE directly interacts with SdhA and FrdA, and is required for the covalent attachment of FAD and the subsequent activation of SDH and FRD (McNeil* et al.*, 2012, 2014). Interestingly, SdhE is conserved in α, β and γ-proteobacteria in addition to eukaryotes, where it is a nuclear-encoded mitochondrial protein termed Sdh5/SdhAF2 (Hao* et al.*, 2009; Huang* et al.*, 2013; Kim & Winge, 2013; McNeil & Fineran, 2013).

Despite our increasing understanding of the function of the widespread SdhE/Sdh5 proteins in the flavinylation and activation of SDH and FRD enzymes, there is a paucity of information about how *sdhE* genes are regulated. Previously, we showed that *sdhE* and *ygfX* were co-transcribed and expressed at similar levels during aerobic or anaerobic growth, which is consistent with both SDH and FRD requiring flavinylation by SdhE (McNeil* et al.*, 2012, 2014). In this study, we show that the DNA-binding master transcriptional activator of flagella biosynthesis, FlhDC, promoted *sdhEygfX* expression. Furthermore, a post -translational anti-FlhDC factor, RsmC (regulator of secondary metabolism C), strongly reduced *sdhEygfX* expression by acting through FlhDC. A post-transcriptional mRNA-binding protein, RsmA (regulator of secondary metabolism A), also reduced *sdhEygfX* levels, but through both FlhDC-dependent and -independent routes. In addition to their role in *sdhEygfX* regulation, RsmA, RsmC and FlhDC exhibited co-ordinate control of motility and prodigiosin production. Consistent with the regulation of *sdhEygfX* by proteins that control metabolism and motility, SdhE controlled metabolism through SDH and FRD (McNeil* et al.*, 2012, 2014), and with FRD was required for maximal flagella-dependent swimming. Finally, to assist our genetic analyses we developed, and describe here, a novel method for genome-editing in bacteria that uses an endogenous type I-F CRISPR (clustered regularly interspaced short palindromic repeats)-Cas (CRISPR associated) system to generate chromosomal point mutations.

## Methods

### Bacterial strains, plasmids and culture conditions.

The bacterial strains and plasmids used in this study are listed in Tables S1 and S2 (available in the online Supplementary Material), respectively. *Serratia* sp. ATCC 39006 (Fineran* et al.*, 2013) and *Escherichia coli* strains were grown at 30 and 37 °C, respectively. Bacteria were grown in lysogeny broth (LB) (5 g yeast extract l^−1^, 10 g bacto tryptone l^−l^ and 5 g NaCl l^−1^), minimal medium [0.1 %, w/v, (NH_4_)_2_SO_4_; 0.41 mM MgSO_4_; 0.2 %, w/v, glucose; 40 mM K_2_HPO_4_; 14.7 mM KH_2_PO_4_; pH 6.9–7.1] at 180 r.p.m., or on LB agar (LBA) (LB supplemented with 1.5 %, w/v, agar) (Miller, 1972). Growth (OD_600_) and absorbance were measured in a Jenway 6300 spectrophotometer. When required, media were supplemented with antibiotics at final concentrations as follow: kanamycin, 50 µg ml^−1^; ampicillin, 100 µg ml^−1^; streptomycin, 50 µg ml^−1^; and chloramphenicol (Cm), 25 µg ml^−1^. Unless noted otherwise, experiments were carried out at least in biological triplicates. For statistical analysis, either one-way ANOVA with a Dunnet post-test, or unpaired *t*-tests, were used. A *P* value less than 0.0001 is indicated by ***, less than 0.001 by ** and less than 0.05 by *.

### Movement of mutations via generalized transduction.

When required, mutations were moved by generalized transduction between strains to generate single, double and triple mutants. For these transductions, phage ΦOT8 was used, as described previously (Evans* et al.*, 2010). The genetic nature of transductants was confirmed by antibiotic-resistance profile and PCR.

### Transposon mutagenesis.

Random transposon mutagenesis of *Serratia *39006 strain HSPIG46 (*sdhEygfX *:: mini-Tn*5lacZ1*) (Fineran* et al.*, 2005b) was performed by conjugation with *E. coli *BW20767 harbouring the Tn-DS1028*uidA *delivery plasmid pDS1028*uidA* (Ramsay* et al.*, 2011). Cultures of the *sdhEygfX *:: mini-Tn*5lacZ1 *mutant and *E. coli *BW20767 donor were grown overnight in LB and 20 µl of each was mixed, pelleted by centrifugation, resuspended in 40 µl LB, spotted onto LBA and incubated for 6 h at 30 °C. Following conjugation, the resulting mating patches were resuspended in 1 ml LB, and 100 µl aliquots of a 1 in 4 dilution were plated onto LBA containing kanamycin, Cm and X-Gal (30 µg ml^−1^). By using this X-Gal screen on plates, transposon mutants were identified that caused altered *sdhEygfX* expression. Transposon insertion mutations were moved into a clean *sdhEygfX *::* lacZ* background via generalised transduction as described above. For quantitative assessment of *sdhEygfX *::* lacZ* expression throughout growth in different mutant backgrounds, standard β-galactosidase assays were used as described previously and expressed as Miller units (MU) (Przybilski* et al.*, 2011).

### Arbitrary PCR.

Transposon insertion sites of mutants of interest were mapped using arbitrary PCR, as described elsewhere (Fineran* et al.*, 2005a; Jacobs* et al.*, 2003). Briefly, PCR was performed on colony DNA using a random primer mix (PF106, PF107 and PF108) and a Tn-DS1028*uidA *specific primer (PF225 or PF338, hybridizing at either end of the transposon). All oligonucleotides used in this study are shown in Table S3. A second PCR was then performed on a 2 µl aliquot of undiluted purified DNA from the first PCR with an adapter primer (PF109) that binds to the 5′ ends of PF106-PF108, and a nested Tn-DS1028*uidA *specific primer (either PF226 or PF294). The resulting mix of PCR fragments was purified and sequenced with the nested Tn-DS1028*uidA *specific primer. The Tn-DS1028*uidA *insertion site and orientation were determined by aligning with the *Serratia *39006 genome (Fineran* et al.*, 2013).

### Phenotypic assays.

Prodigiosin production was assessed as previously described (Slater* et al.*, 2003). For complementation studies of prodigiosin production, plasmid expression was induced at time zero with 1 mM IPTG and assays were performed after 12 h growth. For swimming assays, bacterial cultures were grown overnight in 5 ml LB. The OD_600_ was adjusted to 0.2 and 3 µl was spotted onto tryptic swimming agar (5 g NaCl l^−1^; 10 g tryptone l^−1^; 0.3 %, w/v, agar) plate. Plates were incubated at 30 °C for ~16 h, and swimming measured by the area of the swimming halo. Swarming was assessed as described previously and measured by the swarm area (Williamson* et al.*, 2008). To measure surfactant production, overnight cultures of bacteria were adjusted to an OD_600_ of 0.2 and 5 µl spotted onto LBA plates solidified with 0.75 % (w/v) agar. Plates were incubated for ~16 h at 30 °C and surfactant production determined by the diameter (mm) of the clear ring surrounding the bacterial colony (Williamson* et al.*, 2008). For complementation assays of swimming, swarming and surfactant production, plasmid expression was induced by the addition of 1 mM IPTG to appropriate plates. For β-galactosidase assays on complemented strains, appropriate cultures were grown overnight in 5 ml LB. The OD_600_ was adjusted to give a starting OD_600_ of 0.02 in 25 ml LB with 1 mM IPTG. Strains were grown at 30 °C at 180 r.p.m., and OD_600 _and β-galactosidase activity measured at 12 h.

### Generation of RsmC, RsmA and FlhDC expression plasmids.

Plasmids for expression of RsmA, RsmC and FlhDC were constructed as follows. Firstly, the genes were amplified by PCR using *Serratia* 39006 genomic DNA as the template and primer pairs PF786 and PF787 for *rsmC*, PF788 and PF789 for *rsmA*, and PF796 and PF797 for *flhDC*. The forward primers contained a ribosome-binding site and *Eco*RI sites. The reverse primers contained *Hin*dIII sites, except PF797, which had an *Xma*I site. PCR products were digested with the appropriate enzymes and ligated to pQE-80LoriT that had been previously cut with the same endonucleases. *E. coli* DH5α was transformed with the ligations and plasmids were verified by sequencing. Plasmids were introduced into *Serratia* 39006 by conjugation using either *E. coli* SM10 *λ*pir or S17-1 *λ*pir donors and minimal medium or appropriate antibiotics to counter-select the donors. The nature of the transconjugants was confirmed by antibiotic-resistance testing and PCR.

### Construction of a Δ*flhDC *:: Cm mutant.

The Δ*flhDC *:: Cm deletion plasmid (pPF595) was made by overlap extension PCR. Using *Serratia* 39006 genomic DNA as a template, the left hand fragment contained a 3′ 20 bp sequence that overlapped the 5′ end of the Cm-resistance cassette, whilst the right hand fragment contained a 5′ 20 bp sequence that overlapped the 3′ end of the Cm-resistance cassette. The following primer pairs were used to construct the fragments: left hand fragment, PF817 + PF1289; right hand fragment, PF822 + PF1299. The Cm- resistance cassette was constructed using PF432 + PF433, with pTRB32 as a template. All three fragments were joined using overlap PCR with primers PF817 and PF822. The resulting overlap product was digested with *Bam*HI and *Xba*I, cloned into pBluescript II KS(+) and confirmed by sequencing. Deletion constructs were cloned from pBluescript II KS(+) into pKNG101 using *Bam*HI and *Xba*I. Deletion mutants were generated using an allelic exchange strategy with a sucrose selection protocol similar to that described elsewhere (Fineran* et al.*, 2005a; Kaniga* et al.*, 1991). Putative deletion mutants were sucrose resistant, Cm resistant, non-motile on tryptic swimming agar, and were confirmed by PCR and sequencing.

### Construction of an *flhC* point mutant using endogenous CRISPR-Cas targeting.

The *Serratia* 39006 strain contains a type I-F CRISPR-Cas system (Fineran* et al.*, 2013). A plasmid (pPF704) for the expression of a CRISPR RNA (crRNA) designed to target *flhC* was constructed using primers PF1639 and PF1640. PF1639 had a 19 bp sequence at the 3′ end that overlapped the 3′ end of PF1640. This overlap generated a 106 bp PCR product that contained two repeats of the *Serratia* 39006 type I-F system separated by a 32 bp spacer targeting an internal region of the *flhC* gene. The targeted protospacer region was chosen based on a GG protospacer adjacent motif (PAM) consensus. The product was digested with *Eco*RI and *Sal*I, ligated into pBAD30 and confirmed by sequencing. The vector was used to transform chemically-competent *Serratia* HSPIG46 (*sdhEygfX *:: mini-Tn*5lacZ1*) and Δ*flhDC *:: Cm strains. Following heat shock, cells were recovered in, and plated on, LB and LBA, respectively, both supplemented with glucose (0.2 %, w/v) to repress expression of the targeting plasmid. Transformants containing the targeting plasmid were grown for ~16 h in 5 ml LB with glucose (0.2 %, w/v); 1 ml of this was pelleted and washed twice with PBS to remove any glucose. The pellet was resuspended in 1 ml PBS and then a dilution series was plated onto media containing either glucose (0.2%, w/v) to repress or arabinose (0.1 %, w/v) to induce the crRNA expression required for chromosomal targeting. Surviving colonies that were potential mutants were screened by PCR using PF817 and PF822. One strain was selected for further work and the *flhC*-targeting plasmid was cured by growth without antibiotic selection, resulting in strain PCF185. The *flhC* mutant did not swim and could be complemented with plasmid-encoded *flhDC *(pPF516). Complementation allowed generalized transduction by the flagellum -dependent phage, ΦOT8, of further mutations into the *flhC* mutant. Resulting strains were cured of pPF516.

### Cryo-electron microscopy.

A 4 µl aliquot of overnight culture grown in LB containing the strains analysed [WT and *rsmC*_pro_ (PCF174)] was applied to a glow-discharged Quantifoil 2/2 grid (Quantifoil Micro Tools), blotted and frozen in liquid ethane using a KF80 plunge freezing device (Reichert). Grids with the frozen specimen were loaded into a 914 cryo holder (Gatan) and viewed using a 2200FS cryo transmission electron microscope (JEOL) with an omega filter. Zero-loss images were recorded at microscope magnifications of either ×8000 or ×15 000 using SerialEM software (University of Boulder, Colorado, USA) controlling a TVIPS F416 camera (Tietz Video and Image Processing Systems).

## RESULTS

### Identification of regulators of the *sdhEygfX *operon

To identify genes that affect the expression of *sdhEygfX, *a random transposon mutagenesis was performed in *Serratia* 39006 that contained a chromosomal *sdhEygfX *::* lacZ* transcriptional fusion. Mutants were screened for altered β-galactosidase activity relative to the control strain, and mutants of interest were identified by arbitrary PCR and sequencing. Transposon insertions that affected *sdhEygfX* expression were mapped to two distinct genomic regions – upstream of *rsmA* (regulator of secondary metabolism A) (Williamson* et al.*, 2008), also known as *csrA* (carbon storage regulator A) in *E. coli*, and a gene with similarity to *rsmC* (regulator of secondary metabolism C) from *Pectobacterium carotovorum* and *Pectobacterium atrosepticum* (formerly *Erwinia carotovora* subsp. *carotovora* and subsp. *atroseptica*, respectively) (Cui* et al.*, 1999; Shih* et al.*, 1999). The *rsmC* gene has also been alternatively termed *hexY* (hyperproduction of exoenzymes Y) in *P. atrosepticum* (Bowden* et al.*, 2013; Shih* et al.*, 1999), but is unrelated to the *rsmC* RNA methyltransferase gene of *E. coli*. The analyses of these mutants are discussed in more detail in the following sections.

### RsmA represses *sdhEygfX* expression

**Fig. 1.**
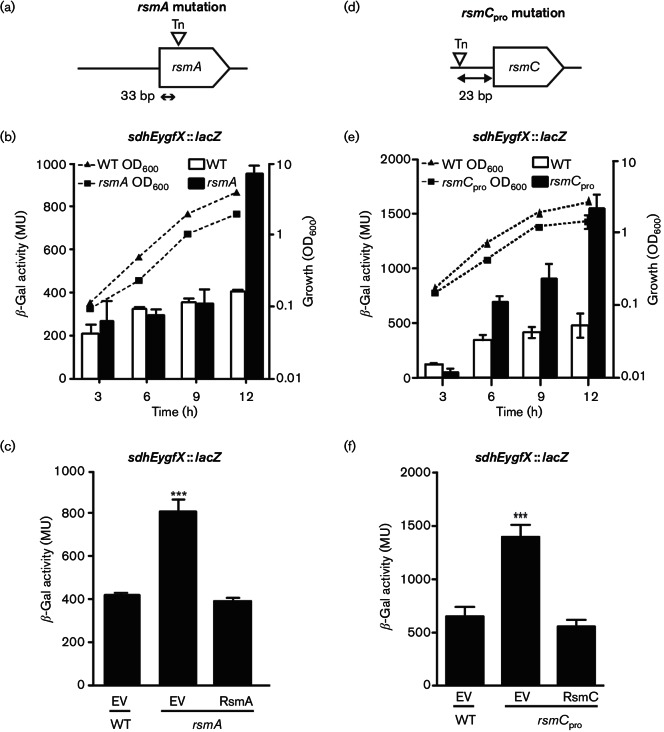
RsmA and RsmC are negative regulators of *sdhEygfX* expression. (a) Schematic of the transposon insertion within *rsmA* (strain NW64). (b) β-Galactosidase activity of the *sdhEygfX *::* lacZ* fusion in a WT strain (HSPIG46) or *rsmA* mutant strain (NW67) background. (c) Complementation of *sdhEygfX *::* lacZ* expression by expression of RsmA (pPF513) or an empty vector (EV) control (pQE-80LoriT) in WT (HSPIG46) or *rsmA* mutant (NW67) backgrounds. (d) Schematic representation of the location of the transposon insertion upstream of *rsmC *(*rsmC*_pro_; strain PCF174). (e) β-Galactosidase activity of the *sdhEygfX *::* lacZ* fusion in a WT background (strain HSPIG46) or in the presence of the *rsmC*_pro_ mutation (strain PCF175). (f) Complementation of *sdhEygfX *::* lacZ* expression by expression of RsmC (pPF512) or an empty vector (EV) control (pQE-80LoriT) in WT (HSPIG46) or the *rsmC*_pro_ mutant (PCF175) backgrounds. Data shown are the means ±  sd (*n*=3). MU, Miller units.

In *Serratia* 39006, RsmA is a pleiotropic regulator, and mutation of *rsmA* results in increased prodigiosin synthesis, swarming and surfactant production (Williamson* et al.*, 2008). One transposon insertion mapped 206 bp upstream of *rsmA* (denoted as *rsmA*_pro_) and caused an increase in *sdhEygfX* expression (Fig. S1). The transposon insertion in the *rsmA*_pro_ mutant disrupted or reduced the production of RsmA, since the increased prodigiosin phenotype was consistent with the elevated pigment in an *rsmA* mutant (Williamson * et al.*, 2008) and could be complemented by plasmid-encoded RsmA (Fig. S2). Independently, we isolated a transposon mutant that mapped within *rsmA *(Fig. 1a). To further investigate the role of *rsmA* in *sdhEygfX* regulation, we used this gene disruption mutant. We constructed an *rsmA, sdhEygfX *::* lacZ *double mutant and assessed *sdhEygfX* expression. The *rsmA* mutation caused up to a >2-fold increase in *sdhEygfX* expression (Fig. 1b). To confirm that the effect of the *rsmA* mutation was due to the absence of RsmA, the strain was complemented by plasmid-encoded RsmA, which restored *sdhEygfX* to levels observed in the WT background containing an empty vector control (Fig. 1c). Previously, we demonstrated increased prodigiosin production when SdhE and YgfX were overexpressed (McNeil* et al.*, 2012, 2013), which is consistent with the elevated *sdhEygfX* expression and pigment levels in *rsmA* mutants (Fig. S2) (Williamson* et al.*, 2008). In conclusion, RsmA negatively affects *sdhEygfX* expression in addition to its roles in secondary metabolism and motility (Wilf* et al.*, 2013; Williamson* et al.*, 2008).

### RsmC represses* sdhEygfX *expression

Two independent transposon insertions were identified upstream of a gene encoding a small 14.5 kDa protein with similarity to RsmC from *Pectobacterium* spp. (Cui* et al.*, 1999; Shih* et al.*, 1999). One mutant with a transposon insertion 23 bp from the translational start of *rsmC* was selected for further work (termed *rsmC*_pro_) (Fig. 1d). In the *rsmC*_pro_ background, *sdhEygfX* expression was increased throughout growth, with up to threefold elevation (Fig. 1e). The increased *sdhEygfX* transcription in the *rsmC*_pro_ background was restored to WT levels by expression of RsmC from a plasmid *in trans* (Fig. 1f), confirming that the transposon insertion had disrupted the synthesis of RsmC. Therefore, RsmC negatively affects *sdhEygfX* expression in *Serratia*.

### RsmC represses prodigiosin synthesis, swimming, swarming and biosurfactant production

**Fig. 2.**
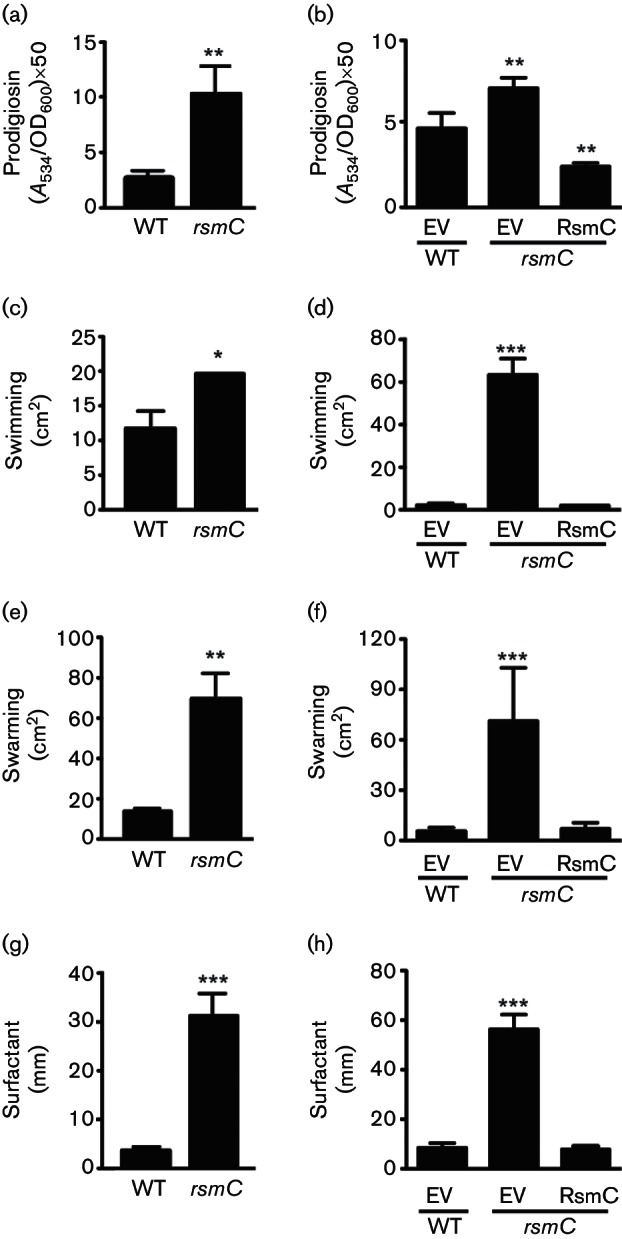
RsmC is a negative regulator of prodigiosin production and motility. (a, b) Prodigiosin production (at 12 h), (c, d) swimming, (e, f) swarming and (g, h) surfactant production in the WT and *rsmC*_pro_ (PCF174) strains. Where relevant, complementation is shown using either an empty vector (EV) control (pQE-80LoriT) or a plasmid expressing RsmC (pPF512). Data shown are the means ± sd (*n*=3).

In *Pectobacterium* spp., *rsmC* mutants are pleiotropic with increased swimming, swarming and production of surfactant and plant cell wall degrading enzymes (Bowden* et al.*, 2013; Chatterjee* et al.*, 2009; Cui* et al.*, 1999, 2008; Shih* et al.*, 1999). However, no study to date has examined the role of *rsmC* outside of the genus *Pectobacterium*. Therefore, we examined prodigiosin synthesis in the *rsmC*_pro_ mutant. The transposon insertion resulted in an approximately fourfold increase in prodigiosin production in the *rsmC* mutant (Fig. 2a). The elevated pigment phenotype was complemented by plasmid-encoded RsmC (Fig. 2b). Thus, RsmC is a newly identified protein involved in the control of prodigiosin synthesis in *Serratia*. Next, the role of RsmC in motility in *Serratia* 39006 was assessed. The *rsmC*_pro_ mutant had increased swimming and swarming compared with that of the WT, and the expression of RsmC *in trans* in the *rsmC*_pro_ mutant complemented these phenotypes (Fig. 2c–f). Swarming in *Serratia* 39006 requires the production of a biosurfactant, the synthesis of which requires RhlA (Williamson* et al.*, 2008). Consistent with the enhanced swarming, biosurfactant production was elevated in the *rsmC*_pro_ mutant compared with that seen in the WT (Fig. 2g) and this effect could be complemented (Fig. 2h). In these complementation assays, the overexpression of RsmC did not significantly affect the OD_600_ when compared with the WT control. The same trend was observed when measuring the ability of surfactant to influence surface tension in drop-collapse assays (Fig. S3).

**Fig. 3.**
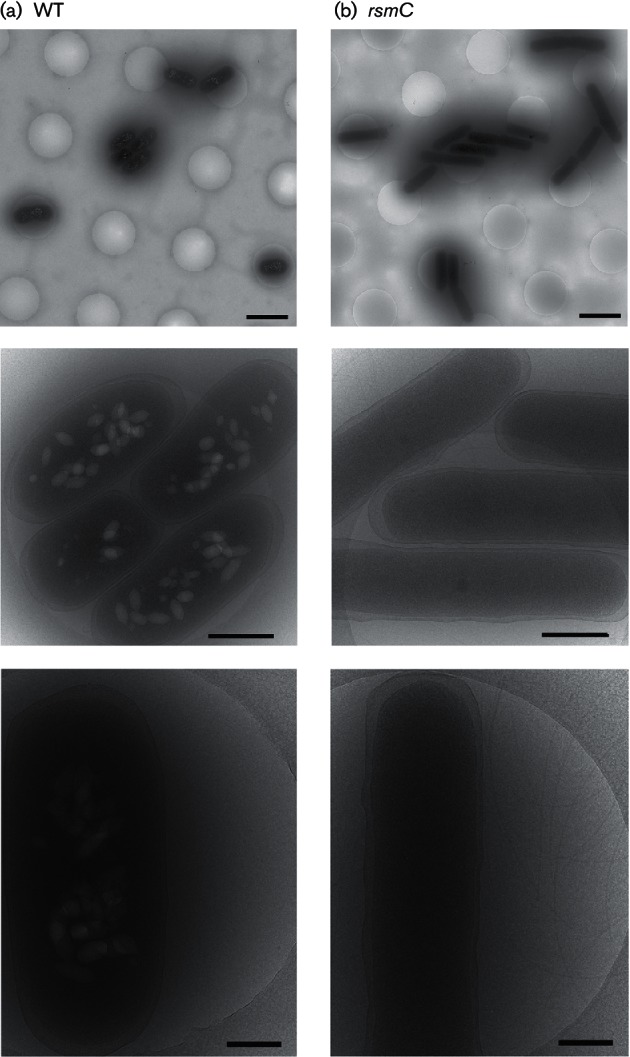
The *rsmC* mutant is elongated, hyper-flagellated and does not contain gas vesicles. Cell morphology by cryo-transmission electron microscopy in the (a) WT and (b) *rsmC*_pro_ (PCF174) strains. Bars, 2 µm (left panel), 500 nm (centre) and 200 nm (right panel). See Fig. S4 for higher resolution images.

Cryo-electron microscopy revealed that the *rsmC*_pro_ mutant was elongated and hyper-flagellated, compared with the WT (Figs 3 and S4) – features typical of swarming cells. We detected abundant gas vesicles in the WT (Fig. 3a), consistent with our earlier work (Ramsay* et al.*, 2011). Gas vesicles are buoyancy organelles that assist bacterial flotation towards air–liquid interfaces in aquatic niches (Ramsay* et al.*, 2011). No gas vesicles were detected in the *rsmC*_pro_ mutant, indicating that RsmC enhances flotation and inhibits swarming (Fig. 3b). In conclusion, RsmC negatively affects prodigiosin synthesis, swimming, swarming and biosurfactant production, and is required for gas vesicle production in *Serratia*.

### FlhDC activates* sdhEygfX *expression

**Fig. 4.**
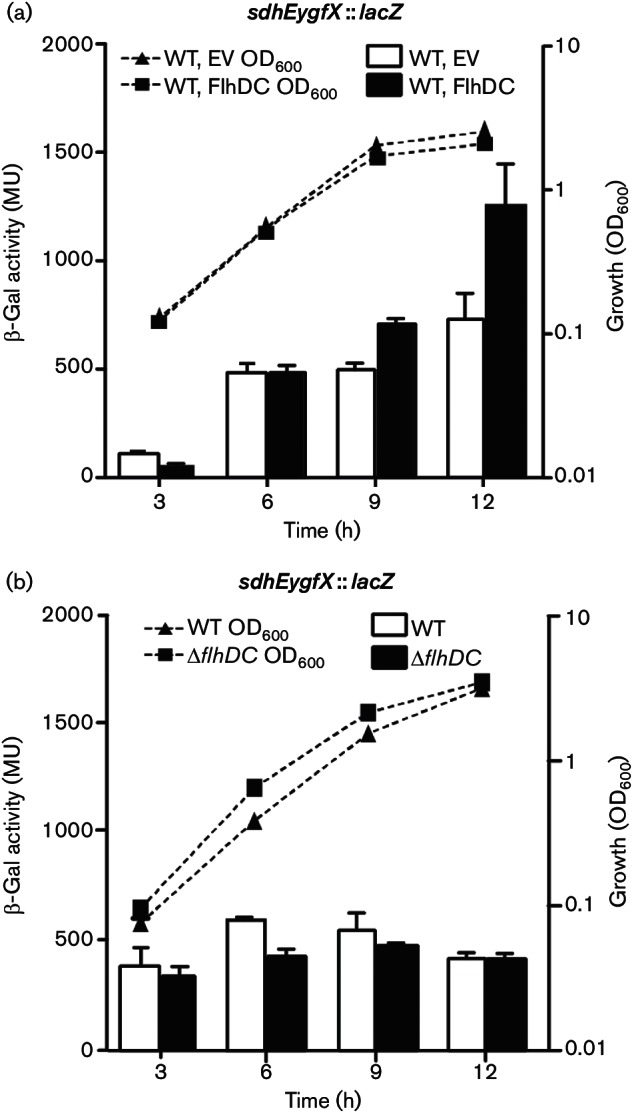
FlhDC activates *sdhEygfX* expression. (a) β-Galactosidase activity of the *sdhEygfX *::* lacZ *fusion in a WT background (strain HSPIG46) with either an empty vector (pQE-80LoriT) or FlhDC (pPF516). (b) β-Galactosidase activity of the *sdhEygfX *::* lacZ *fusion in a WT background (strain HSPIG46) or in the *flhDC *mutant (Δ*flhDC *:: Cm). Data shown are the means ± sd (*n*=3).

A common feature of RsmA and RsmC is that they control the master regulator of flagella biosynthesis (FlhDC) (Chatterjee * et al.*, 2009; Williamson* et al.*, 2008). In Enterobacteriaceae, the FlhD_4_C_2 _complex is produced from the *flhDC* operon, and activates a cascade of flagellar and chemotaxis gene expression (Chevance & Hughes, 2008). In *Serratia* 39006, *rsmA* mutants have elevated levels of the *flhDC* regulator and *rhlA* biosurfactant mRNAs (Wilf* et al.*, 2013; Williamson* et al.*, 2008). To control motility in *Pectobacterium*, RsmC directly interacts with, and inhibits, the FlhDC protein complex (Chatterjee* et al.*, 2009). Therefore, we hypothesized that RsmA and RsmC may act through FlhDC, and that this master regulator could affect *sdhEygfX* expression. Indeed, expression of FlhDC from a plasmid *in trans* elevated *sdhEygfX* transcription (Fig. 4a), demonstrating that FlhDC activates *sdhEygfX*. In contrast, deletion of *flhDC* caused only a subtle reduction, if any, in *sdhEygfX* expression (Fig. 4b). Since both RsmA and RsmC inhibit FlhDC (Chatterjee* et al.*, 2009; Williamson* et al.*, 2008), there is very little active FlhDC in the WT background during growth in broth, which is likely to explain the stronger effect on *sdhEygfX* caused by FlhDC overexpression (Fig. 4a). We could not identify a putative FlhDC-binding site upstream of *sdhEygfX*, suggesting that FlhDC activation is indirect. In summary, FlhDC activates *sdhEygfX* expression.

### FlhDC activates prodigiosin, swimming, swarming and biosurfactant production

**Fig. 5.**
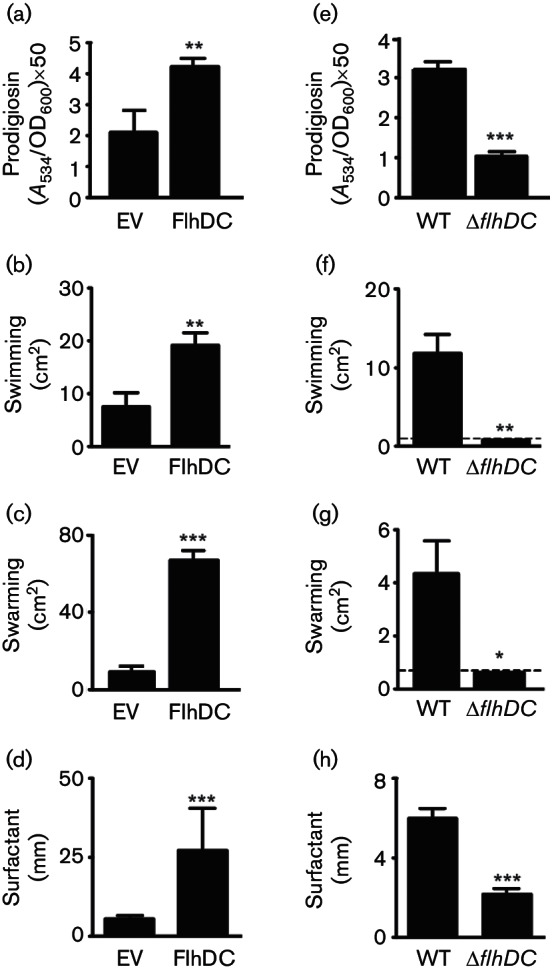
FlhDC activates prodigiosin production and motility. (a) Prodigiosin production (at 12 h), (b) swimming, (c) swarming and (d) surfactant production in a WT with either an empty vector (EV; pQE-80LoriT) or a plasmid expressing FlhDC (pPF516). (e) Prodigiosin production, (f) swimming, (g) swarming and (h) surfactant production in the WT or in the *flhDC *mutant (Δ*flhDC *:: Cm). Data shown are the means ± sd (*n*=3). Where shown, dashed lines represent the limits of detection.

Since RsmA and RsmC inhibit prodigiosin synthesis and motility, we examined the role of FlhDC on these phenotypes in *Serratia* 39006. Expression of plasmid-encoded FlhDC resulted in increased prodigiosin biosynthesis, swimming, swarming and surfactant production in the WT background (Fig. 5a–d) – phenotypes associated with *rsmA* and *rsmC* deletion. In reciprocal experiments, deletion of *flhDC* caused the opposite effects, a decrease in pigment production, and swimming, swarming and biosurfactant synthesis were undetectable (Fig. 5e–h). Therefore, FlhDC activates prodigiosin production and motility. The role of FlhDC in regulating both motility and *sdhEygfX* suggested that SdhE might play a part in motility. Deletion of *sdhE* resulted in reduced swimming compared with the WT (Fig. S5), whereas *ygfX* had no discernable effect (McNeil* et al.*, 2012). In *E. coli*, FRD associates with the flagella switch complex and is required for aerobic motility (Cohen-Ben-Lulu* et al.*, 2008). In *Serratia* 39006, *frdABCD* mRNA is detected during aerobic growth (Wilf* et al.*, 2013), so we hypothesized that SdhE activates FRD to influence swimming. Indeed, maximal swimming required both FRD and SdhE (Fig. S5), most likely due to SdhE-dependent flavin ylation of FrdA and activation of FRD (McNeil* et al.*, 2014).

### Generation of a missense *flhC* mutant using endogenous type I-F CRISPR-Cas targeting

**Fig. 6.**
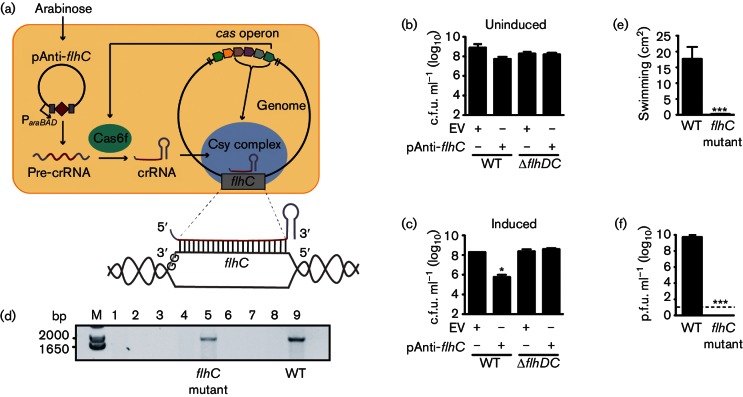
Generation of an *flhC* mutant using endogenous CRISPR-Cas targeting. (a) Schematic of the pAnti-*flhC* plasmid (pPF704) and the endogenous type I-F CRISPR-Cas system in *Serratia* 39006. (b, c) Number of c.f.u. of WT or Δ*flhDC *:: Cm strains containing the pAnti-*flhC* plasmid (pPF704) in (b) uninduced and (c) induced conditions. (d) PCR screening of the *flhDC* locus using primers PF817 and PF822. Empty lanes are due to strains with deletions larger than the *flhDC* operon. The band in the *flhC* mutant lane is the same size as the band in the WT control lane, but contains a GG to GA PAM mutation. (e, f) Swimming (e) and ΦOT8 phage infection (f) of the resulting *flhC* mutant (PCF185). The dashed line in (f) represents the limit of detection. Data shown are the means ± sd (*n*=3).

To test whether the reduced *sdhEygfX* expression elicited by RsmA and RsmC required FlhDC, double and triple mutants were required. Since the *flhDC, rsmA* and *rsmC* mutants had the same resistance markers, we made an unmarked *flhC *mutant. The construction of markerless allelic exchange mutants in *Serratia* 39006 can be inefficient; therefore, we developed a new method based on CRISPR-Cas genome-editing. CRISPR-Cas systems are bacterial adaptive immune systems that use small RNAs to guide protein complexes to complementary DNA and cause cleavage (Richter* et al.*, 2012a). We previously showed that a strain with an existing type I-F CRISPR-Cas system could be exploited to generate large deletion mutations in the host chromosome (Dy* et al.*, 2013; Vercoe* et al.*, 2013). To exploit the type I-F CRISPR-Cas system present in *Serratia* 39006, a plasmid was generated with an inducible guide crRNA (a short 32 bp spacer sequence matching an internal region of *flhC*) between two 28 bp type I-F repeats (Fig. 6a) (Fineran* et al.*, 2013). The targeted region (termed a protospacer) was flanked by a GG PAM required for DNA cleavage (Almendros* et al.*, 2012; Vercoe* et al.*, 2013). The strategy relied on expression of the repeat–spacer–repeat RNA (termed a precursor-crRNA), crRNA generation by the host Cas6f (Przybilski* et al.*, 2011) and formation of an endogenous Csy interference complex (Richter* et al.*, 2012b; Wiedenheft* et al.*, 2011). The interference complex should then target chromosomal *flhC*, causing cell death and enabling the selection of *flhC* mutants that escape targeting (Vercoe* et al.*, 2013).

The anti-*flhC* plasmid was induced in the *sdhEygfX *::* lacZ *background and a >100-fold reduction in viable count was detected compared with controls (Fig. 6b, c). To demonstrate targeting of *flhC*, the experiments were performed in the Δ*flhDC* strain that lacks the *flhC* target. Consistent with specific targeting, no reduction in viable count was observed in this strain (Fig. 6b, c). Survivors following genome-targeting were screened for the *flhDC* region by PCR. Of the ~500 colonies screened, the majority had deletions larger than the *flhDC* operon. This is consistent with our earlier study in *P. atrosepticum*, where large deletions of ~100 kb resulted from chromosomal targeting (Vercoe* et al.*, 2013). Other mutations, such as those within the PAM or protospacer, allow escape from targeting (Fineran, *et al.*, 2014). Indeed, three mutants with an *flhDC* locus of WT size (example in Fig. 6d) were sequenced and contained a GG to GA PAM substitution, resulting in a missense FlhC A24V mutation. The targeting plasmid was cured from one strain. The FlhC A24V mutant was non-motile (Fig. 6e) and, as expected, resistant to the flagellum-dependent phage ΦOT8 (Fig. 6f) (Evans* et al.*, 2010). To our knowledge, this generation of an unmarked *flhC* mutant is the first demonstration that endogenous type I-F CRISPR-Cas systems can be used to generate point mutants in bacterial chromosomes.

### RsmA and RsmC repress* sdhEygfX *expression via FlhDC

**Fig. 7.**
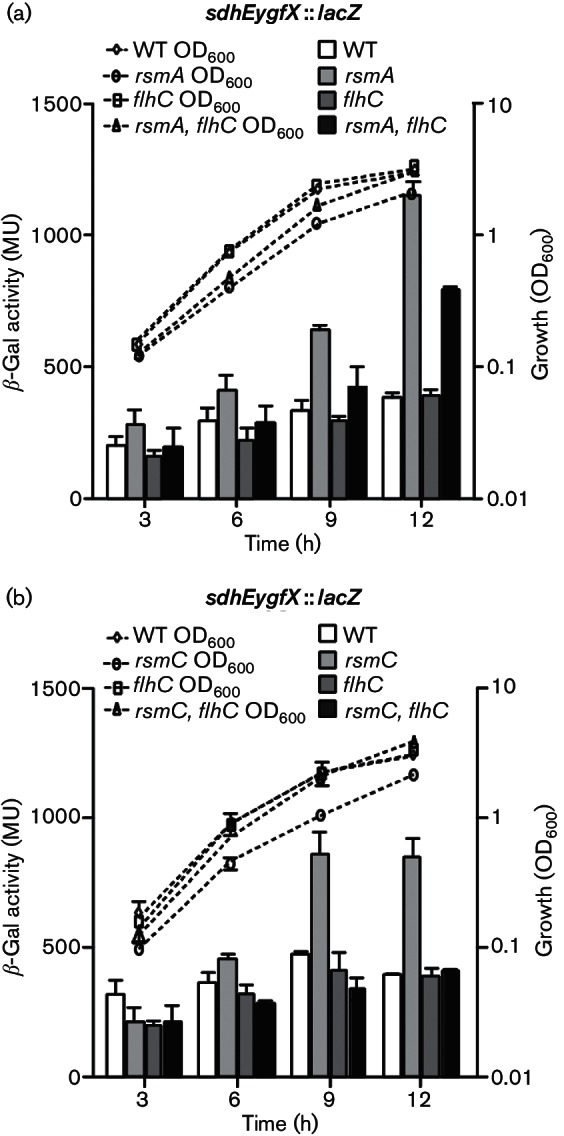
RsmA and RsmC repress *sdhEygfX* transcription via *flhDC*. (a) The β-galactosidase activity of the *sdhEygfX *::* lacZ* fusion was assessed in WT (HSPIG46), *rsmA* (NW67), *flhC* (PCF185) and *rsmA, flhC* (PCF186) backgrounds. (b) The β-galactosidase activity of the *sdhEygfX *::* lacZ* fusion was assessed in WT (HSPIG46), *rsmC*_pro_ (PCF175), *flhC* (PCF185) and *rsmC*_pro_*, flhC* (PCF187) backgrounds. Data shown are the means ± sd (*n*=3).

To determine whether the RsmC- and RsmA-dependent regulation of *sdhEygfX* acted through FlhDC, *sdhEygfX* transcription was assessed in various mutant backgrounds. As observed previously, mutation of *rsmA* led to increased *sdhEygfX* expression (Fig. 7a). However, mutation of *flhC* in the *rsmA *mutant partially abolished the RsmA-dependent repression of *sdhEygfX* expression seen in the single *rsmA* mutant (Fig. 7a). Therefore, RsmA negatively regulates *sdhEygfX* in an FlhDC-dependent manner (Wilf* et al.*, 2013; Williamson* et al.*, 2008), while also inhibiting *sdhEygfX* via an FlhDC-independent pathway. The FlhDC-dependent pathway is supported by our recent RNA sequencing (RNA-seq) and quantitative reverse transcription-PCR analyses of an *rsmA* mutant, which revealed increased *flhDC* mRNA and mRNAs encoding other flagella proteins in the *rsmA* mutant compared with the WT (Wilf* et al.*, 2013; Williamson* et al.*, 2008).

The *rsmC* mutation resulted in increased *sdhEygfX* transcription that was entirely FlhDC-dependent (Fig. 7b). Importantly, mutation of *flhC* in the *rsmC* background caused the elevated *sdhEygfX* expression in the single *rsmC* mutant to return to levels observed in both the WT and *flhC* mutant (Fig. 7b). These observations are supported by previous work in *P. carotovorum*, where an *rsmC* mutation had no effect in an *flhDC* mutant (Chatterjee* et al.*, 2009). In conclusion, the regulation of the *sdhEyfgX* operon by RsmC occurs in an FlhDC-dependent manner, whereas RsmA has both FlhDC-dependent and -independent effects on *sdhEyfgX *transcription.

## DISCUSSION

**Fig. 8.**
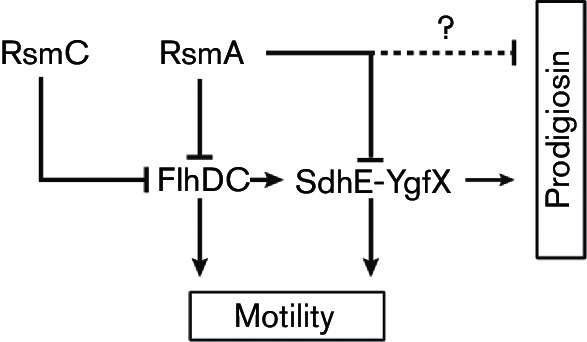
Proposed model of regulation of *sdhEygfX*. RsmC binds FlhDC and inhibits its activity. RsmA negatively affects the levels of the *flhDC* mRNA and transcription of *sdhEygfX*. FlhDC activates the transcription of *sdhEygfX*, which promotes prodigiosin production and motility. FlhDC also affects motility independently of SdhE-YgfX and the pleiotropic regulator RsmA is also likely to function via additional pathways (dotted line).

In this study, we have investigated the regulation of the *sdhEygfX *operon in *Serratia* 39006. We identified an overlapping pathway involving the post-transcriptional regulators RsmA and RsmC which repressed *sdhEygfX* expression by acting through the flagella master regulatory complex, FlhDC. FlhDC activated *sdhEygfX* transcription and the inhibitory effect of RsmC was dependent on *flhDC*. In contrast, RsmA repressed *sdhEygfX* in both an FlhDC-dependent and -independent manner (Fig. 8). Currently, it is not known how SdhE and YgfX influence pigment production. SdhE was initially identified as a gene neighbouring YgfX, a regulator of prodigiosin production, and the deletion of either, or both, of these genes results in a decrease in transcription of the prodigiosin biosynthesis operon (McNeil* et al.*, 2012). The physiological role of prodigiosin has been an issue of debate (Williamson* et al.*, 2006), but previous work uncovered an antibiotic effect that is elicited in a surfactant-dependent manner (Williamson* et al.*, 2008). This led to a model whereby swarming and surfactant production may enable the local dispersal of the prodigiosin antibiotic to help *Serratia* in niche colonization and competition with other bacteria (Williamson* et al.*, 2008). Our data show that RsmA, RsmC and FlhDC co-ordinately regulate motility, surfactant and antibiotic pigment production, which is consistent with the synergism between biosurfactant and prodigiosin (Williamson* et al.*, 2008).

RsmA is a homologue of CsrA (carbon storage regulator) from *E. coli*, which is a post-transcriptional regulator that binds to the 5′ untranslated regions of mRNA and either represses translation (by occluding ribosome-binding sites) or stabilizes transcripts by blocking RNase E-dependent cleavage (Romeo* et al.*, 2013; Vakulskas* et al.*, 2015). As its nomenclature implies, CsrA affects carbon flux, but it is also a highly pleiotropic regulator that controls other processes, including motility and virulence. The mRNA-binding activity of CsrA can be out-competed by small antagonistic RNAs (CsrB and CsrC) that fold into secondary structures generating binding sites in the single-stranded loops that sequester the CsrA partner (Romeo* et al.*, 2013; Vakulskas* et al.*, 2015). In *Serratia* 39006, *rsmA* mutants exhibit enhanced prodigiosin production, swarming and biosurfactant production (Williamson* et al.*, 2008). Indeed, in an *rsmA* mutant the mRNA involved in biosurfactant synthesis (*rhlA*) was increased by ~60-fold and transcripts of the *flhDC* operon were ~8-fold higher than in the WT strain (Williamson* et al.*, 2008). Furthermore, a recent RNA-seq and proteomic study in *Serratia* 39006 showed that an *rsmA* mutant produced increased flagellar components and many prodigiosin biosynthetic proteins were elevated (Wilf* et al.*, 2013). Our *rsmA* data are consistent with these studies, but also demonstrate another route for RsmA and FlhDC (via *sdhEygfX*), by which additional control of metabolism (via SDH and FRD; McNeil* et al.*, 2012, 2014) and motility may be modulated. Both *sdhE* and FRD mutants show reduced swimming, which echoes results with *E. coli, *where FRD binds to the flagellar switch, thereby impacting flagellar assembly and switching (Cohen-Ben-Lulu* et al.*, 2008). Adjusting SdhE levels in response to different regulatory cues should allow the bacterium to ensure appropriate flavin ylation /activation of FRD to fine-tune motility.

To our knowledge, this is the first study of RsmC outside of the genus *Pectobacterium*. RsmC is exclusive to Enterobacteriaceae, being mainly present in the genera *Pectobacterium* and *Dickeya*. However, some homologues exist in other genera (e.g. *Brenneria* and *Lonsdalea*). Despite its name, RsmC is not a bona fide member of the Rsm pathway, but controls some shared phenotypes. In *Pectobacterium* spp., RsmC directly binds FlhDC and antagonizes its function (Chatterjee* et al.*, 2009). Mutation of *rsmC* causes increased swimming, swarming and production of surfactant and plant cell wall degrading enzymes (Bowden* et al.*, 2013; Chatterjee* et al.*, 2009; Cui* et al.*, 1999, 2008; Shih* et al.*, 1999). In agreement, RsmC repressed swimming, biosurfactant production and swarming in *Serratia* 39006. The *Serratia * 39006 *rsmC* mutant also produced longer, hyper-flagellated cells, but no gas vesicles. This shows that RsmC (and by inference FlhDC) inversely controls swimming and floatation. Similarly, RsmA displays inverse control of swimming and gas vesicle morphogenesis (Ramsay* et al.*, 2011), which conceivably might be occurring via FlhDC. We also provide the first evidence to our knowledge that RsmC and FlhDC differentially affect prodigiosin production. Interestingly, an earlier study reported that flagellin protein variation correlated with pigment variation in *Serratia marcescens* (Paruchuri & Harshey, 1987).

The signals that regulate this *sdhEygfX *control pathway are currently unknown. However, it is likely that RsmA and RsmC inhibit FlhDC to reduce the expression of *sdhEygfX* under conditions where motility, prodigiosin or maximal SDH or FRD activity are not required. Importantly, *sdhEygfX* expression is still robust, even in the absence of FlhDC, and under both aerobic and anaerobic conditions, ensuring sufficient active SDH and FRD for metabolism (McNeil* et al.*, 2014). The non-coding RNA antagonists of RsmA proteins are activated by the GacAS two-component signalling systems (Romeo* et al.*, 2013; Vakulskas* et al.*, 2015). The signals for GacAS systems appear to be intermediates of carbon metabolism, including acetate, and GacAS responds to intracellular levels of TCA cycle intermediates (α-ketoglutarate, succinate and fumarate) that we predict should signal increased activation of the associated metabolic pathways (Chavez* et al.*, 2010; Takeuchi* et al.*, 2009). Thus, it is possible that in *Serratia*, these TCA precursors/intermediates would up-regulate RsmB via GacAS signalling (PigQW in *Serratia*; Fineran* et al.*, 2005b; Williamson* et al.*, 2008). RsmB would sequester RsmA and lead to elevated *sdhEygfX* (in FlhDC-dependent and -independent pathways). The increased SdhE would ensure activation of the TCA cycle and the electron transport chain through SDH and/or FRD flavinylation to support metabolism (McNeil* et al.*, 2012, 2014). To date, it is not known what regulates RsmC.

Here, we have also developed and demonstrated the feasibility of using endogenous CRISPR-Cas targeting by type I systems to isolate point mutations in target genes. This is an extension of our previous work, which showed that large regions, such as entire pathogenicity islands, could be deleted (Vercoe* et al.*, 2013). Despite the widespread uptake of Cas9 genome-editing in eukaryotes, few studies have explored CRISPR-Cas utility in bacteria (Selle & Barrangou, 2015) and almost all use the Cas9 technology (Cobb* et al.*, 2015; Jiang* et al.*, 2013; Li* et al.*, 2015; Oh & van Pijkeren, 2014; Tong* et al.*, 2015). The simplicity of the CRISPR-Cas9 system, and its ability to make double-stranded breaks without further degradation, make it the favoured CRISPR-Cas type for genome-editing. However, in those bacteria with few, or no, current genetic tools, exploiting endogenous CRISPR-Cas systems has considerable potential (Selle & Barrangou, 2015; Vercoe* et al.*, 2013). For applications of endogenous CRISPR-Cas systems, type I are the most abundant and well characterized. A distinction from CRISPR-Cas9 is that the processive DNA degradation caused by Cas3 in type I systems typically causes large deletions (Vercoe* et al.*, 2013). Indeed, the vast majority of mutants we generated in this study contained deletions of the *flhDC* region. Developing methods to control the extent of deletions, either through mutagenesis of Cas proteins or by providing substrates for homology-directed repair, is essential to harness the potential of genome-editing and gene silencing using type I CRISPR-Cas systems (Fineran & Dy, 2014; Selle & Barrangou, 2015). Nevertheless, in this study, we successfully isolated three point mutations in *flhC* and so our results suggest strongly that further refinement of this CRISPR-Cas-based approach to bacterial mutagenesis could have a generic utility for precise engineering of prokaryotes – with implications from basic microbiology through synthetic biology to industrial, agricultural and medical translation.

## Supplementary Data

354 Supplementary File 1
