# Supplementary material for: CRISPR-Cas gene-editing reveals RsmA and RsmC act through FlhDC to repress the SdhE flavinylation factor and control motility and prodigiosin production in Serratia
Source: Microbiology (Reading). 2016 Jun;162(6):1047–58. doi: 10.1099/mic.0.000283 (PMC5042078; doi:10.1099/mic.0.000283)
Supplement: Supplementary file 1 [file mic-162-1047-s001.pdf]

SUPPLEMENTARY MATERIAL

CRISPR-Cas gene editing reveals RsmA and RsmC act through FlhDC to repress the SdhE flavinylation factor and control motility and prodigiosin production in *Serratia*

Hannah G. Hampton[1,§], Matthew B. McNeil[1,#,§], Thomas J. Paterson[1], Blair Ney[1,#], Neil R. Williamson[2], Richard A. Easingwood[3], Mihnea Bostina[1,3], George P.C. Salmond[2] and Peter C. Fineran[1]*

[1]Department of Microbiology and Immunology, University of Otago, PO Box 56, Dunedin 9054, New Zealand. [2]Department of Biochemistry, University of Cambridge, Tennis Court Road, Cambridge CB2 1QW, UK. [3]Otago Centre for Electron Microscopy, University of Otago, PO Box 56, Dunedin 9054, New Zealand.

[#]Present addresses: MBM, Infectious Disease Research Institute, Seattle, Washington, United States of America. BN, Faculty of Education, Science, Technology & Mathematics, University of Canberra, ACT 2601, Australia.

[§]These authors contributed equally to this work.

*For correspondence. E-mail. peter.fineran@otago.ac.nz; Tel. +64 (0)3 479 7735; Fax. +64 (0)3 479 8540.

Table S1. Strains used in this study

Strain	Genotype/Phenotype	Reference
Escherichia coli		
CC118 λ pir	*araD, Δ(ara, leu), ΔlacZ74, phoA20, galK, thi-1, rspE, rpoB, argE, recA1, λ pir*	(Herrero *et al.*, 1990)
DH5 α	F-, Δ80Δd*lacZ*M15, Δ (*lacZYA–argF*)*U169*, *endA1*, *recA1*, *hsdR17* ($r_K^- m_K^+$), *deoR*, *thi-1, supE44*, λ $^-$, *gyrA96*, *relA1*	Gibco/BRL
HH26	Marker exchange mobilization strain for conjugal transfer	(Grinter, 1983)
S17-1 λ pir	*recA*, *pro*, *hsdR*, *recA*::RP4-2-Tc::Mu, λ *pir*, Tmp^R, Sp^R, Sm^R	(de Lorenzo *et al.*, 1990)
SM10 λ pir	*thi-1, thr, leu, tonA, lacY, supE, recA*::RP4-2-Tc::Mu, λ pir, Km^R	(de Lorenzo *et al.*, 1990)
***Serratia* sp. ATCC 39006** *(all in LacA background)*		
LacA (referred to as "WT" in this study)	Lac^- derivative of *Serratia* sp. ATCC 39006	(Thomson *et al.*, 2000)
Δ*sdhE*	Markerless deletion mutant of *sdhE*	(McNeil *et al.*, 2012)
Δ*frdABCD*::Cm	Deletion mutant of *frdABCD* replaced with Cm^R	(McNeil *et al.*, 2014)
Δ*sdhE*, Δ*frdABCD*::Cm	*sdhE* and *frdABCD* double mutant constructed via transduction of Δ*frdABCD*::Km into Δ*sdhE*, Km^R	(McNeil *et al.*, 2014)
Δ*flhDC*::Cm	Deletion mutant of *flhDC* replaced with a Cm^R	This study
Δ*flhDC*::Cm, *sdhEygfX*::*lacZ*	Δ*flhDC*::Cm, *sdhEygfX*::mini-Tn5*lacZ1*, constructed via transduction of *sdhEygfX*::mini-Tn5*lacZ1* into Δ*flhDC*::Cm, Cm^R, Km^R	This study
HSPIG46	*sdhEygfX*::mini-Tn5*lacZ1*, Km^R	(Fineran *et al.*, 2005)
NW64	*rsmA*::Tn-DS1028*uidA*, Cm^R	This study
NW67	*rsmA*::Tn-DS1028*uidA*, *sdhEygfX*::mini-Tn5*lacZ1*, Cm^R, Km^R; Tn insertion from NW64 transduced into HSPIG46	This study
PCF174	*rsmC*$_{pro}$::Tn-DS1028*uidA*, Cm^R; Tn insertion from original mutant transduced into LacA	This study
PCF175	*rsmC*$_{pro}$::Tn-DS1028*uidA*, *sdhEygfX*::mini-Tn5*lacZ1*, Cm^R, Km^R; Tn insertion from PCF174 transduced into HSPIG46	This study
PCF176	*rsmA*$_{pro}$::Tn-DS1028*uidA*, Cm^R; Tn insertion from original mutant transduced into LacA	This study
PCF177	*rsmA*$_{pro}$::Tn-DS1028*uidA*, *sdhEygfX*::mini-Tn5*lacZ1*, Cm^R, Km^R; Tn insertion from PCF176 transduced into HSPIG46	This study
PCF185	*flhC* mutant, *sdhEygfX*::mini-Tn5*lacZ1,* Km^R	This study
PCF186	*flhC* mutant, *rsmA*::Tn-DS1028*uidA*, *sdhEygfX*::mini-Tn5*lacZ1*, Cm^R, Km^R; Tn insertion from NW64 transduced into PCF185	This study
PCF187	*flhC* mutant, *rsmC*$_{pro}$*::*Tn-DS1028*uidA, sdhEygfX*::mini-Tn5*lacZ1*, Cm^R, Km^R	This study

Table S2. Plasmids used in this study

Plasmid Name	Description	Reference
pBAD30	Arabinose inducible expression vector, p15A replicon, Ap^R.	(Guzman *et al.*, 1995)
pBluescript II KS(+)	Cloning vector, ColE1 replicon, Ap^R	Stratagene
pDS1028uidA	Conjugative plasmid containing Tn-DS1028*uidA* (mini-Tn*5*-based), oriR6K, Cm^R, Tc^R	(Ramsay *et al.*, 2011)
pKNG101	Marker exchange suicide vector, *sacBR*, mobRK2, oriR6K, Sm^R	(Kaniga *et al.*, 1991)
pNJ5000	Mobilizing plasmid used in marker exchange, Tc^R	(Grinter, 1983)
pPF512	pQE-80LoriT containing *rsmC*, Ap^R	This study
pPF513	pQE-80LoriT containing *rsmA*, Ap^R	This study
pPF516	pQE-80LoriT containing *flhDC*, Ap^R	This study
pPF595	pBluescript II KS(+) with the Δ*flhDC*::Cm construct, Ap^R	This study
pPF596	pKNG101 with the Δ*flhDC*::Cm construct, Sm^R	This study
pPF704	pBAD30 containing *flhC* targeting fragment, Ap^R	This study
pQE-80LoriT	Expression vector derivative of pQE-80L with RP4 *oriT*, Ap^R	(Gristwood *et al.*, 2011)
pTRB30	Expression vector derivative of pQE-80L, Km^R	(Przybilski *et al.*, 2011)
pTRB32	Expression vector derivative of pQE-80L, Cm^R	Tim Blower; unpublished

Table S3. Oligonucleotides used in this study

Primer Name	Sequence (5’-3’)	Notes	Restriction sites (bold)
PF106	GACCACACGTCGACTAGTGCNNNNNNNNNNAGAG	Arbitrary PCR primer 1	
PF107	GACCACACGTCGACTAGTGCNNNNNNNNNNACGCC	Arbitrary PCR primer 2	
PF108	GACCACACGTCGACTAGTGCNNNNNNNNNNGATAC	Arbitrary PCR primer 3	
PF109	GACCACACGTCGACTAGTGC	Arbitrary PCR adapter primer	
PF209	TCGTCTTCACCTCGAGAAATC	F pQE-80LoriT MCS	
PF210	GTCATTACTGGATCTATCAACAGG	R pQE-80LoriT MCS	
PF213	CAACTTAACGTAAAAACAACTTCAGA	F pKNG101 MCS	
PF214	TACACTTCCGCTCAGGTCCTTGTCCT	R pKNG101 MCS	
PF217	CGACGTAAAACGACGGCCAGT	F pBluescript II (KS)+ MCS	
PF218	GGAAACAGCTATGACCATG	R pBluescript II (KS)+ MCS	
PF225	GATAATAAGCGGATGAATGGCAG	Tn-DS1028*uidA* out RHS	
PF226	CATAAGGGACTCCTCATTAAG	Tn-DS1028*uidA* nested primer out LHS	
PF294	CTTGCTCAATCAATCACCG	Tn-DS1028*uidA* nested primer out RHS	
PF338	ATCTGCATCGGCGAACTGAT	Tn-DS1028*uidA* out LHS	
PF432	TTT**GTCGAC**ATACCGGGAAGCCCTGGG	R Cm^R cassette	SalI
PF433	TTT**AAGCTT**AGGCGTTTAAGGGCACCA	F Cm^R cassette	HindIII
PF786	ATA**GAATTC**AGGAGGAATATAATGAGTCTGATATTTGGGCAG	F *rsmC* with RBS	EcoRI
PF787	GAT**AAGCTT**TTAAGAAGCGAGGTGTGATG	R *rsmC*	HindIII
PF788	ATA**GAATTC**AGGAGGAATATAATGCTTATTTTAACTCGTCG	F *rsmA* with RBS	EcoRI
PF789	GAT**AAGCTT**TCAATAAGATGTTGGCTGAG	R *rsmA*,	HindIII
PF796	ATA**GAATTC**AGGAGGAATATAATGGGTACTTCTGAG TTACTTAAGC	F *flhDC* with RBS	EcoRI
PF797	GAT**CCCGGG**TCAGACTGCGTGTTTTACTTG	R *flhDC*	XmaI
PF817	ATA**GGATCC**TTCGTAATTGATAAGTGGTTTG	F Δ*flhDC*::Cm LHF	BamHI
PF822	GAT**TCTAGA**AATTCAACAATATAAGCGTCTG	R Δ*flhDC*::Cm RHF	Xba1
PF1298	ACGCCTAAGCTTAAAAATCTCATCCCGGCAAG	R Δ*flhDC*::Cm LHF	
PF1299	CGGTATGTCGACAAAGCCTGGTAGCGATTTATAAG	F Δ*flhDC*::Cm RHF	
PF1639	TTT**GAATTC**GTTCACTGCCGTACAGGCAGCTTAGAAA*CGCCTGCAGATGCTCGAAAGTG*	F for *flhC* targeting (repeat underlined, partial spacer italic)	EcoRI
PF1640	AAA**GTCGAC**TTTCTAAGCTGCCTGTACGGCAGTGAAC*AACTGCGTTTCACTTTCGAGCATCTGCAG*	R for *flhC* targeting spacer (repeat underlined, partial spacer italic)	SalI

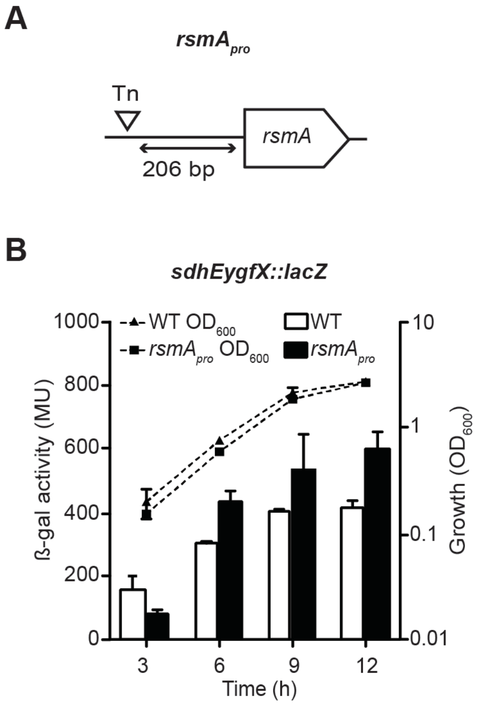

Figure S1. RsmA is a negative regulator of *sdhEygfX* expression. A. Schematic representation of the location of the transposon insertion upstream of *rsmA* (*rsmA*$_{pro}$; strain PCF176). **B.** β-galactosidase activity of the *sdhEygfX*::*lacZ* fusion in a WT background (strain HSPIG46) or in the presence of the *rsmA*$_{pro}$ mutation (strain PCF177). Data shown are the means ± SD (*n*=3).

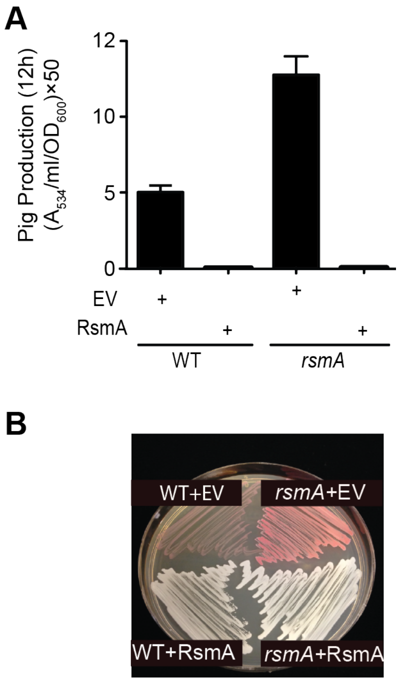

Figure S2. Prodigiosin production and complementation in the *rsmA*$_{\text{pro}}$ mutant. A Prodigiosin assay and **B** photo of prodigiosin production in WT and *rsmA*$_{\text{pro}}$ strain (PCF176) containing either an empty vector control (EV; pQE-80LoriT) or a plasmid expressing RsmA (pPF513). In (A) data shown are the means ± SD (*n*=3).

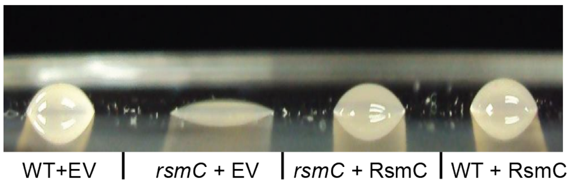

Figure S3. RsmC represses surfactant production. Drop collapse assay that qualitatively measures the surface tension effects of surfactant production in WT and $rsmC_{pro}$ (strain PCF174) containing either an empty vector control (EV; pQE-80LoriT) or a plasmid expressing RsmC (pPF512). The assay was performed as described previously (Williamson *et al.*, 2008).

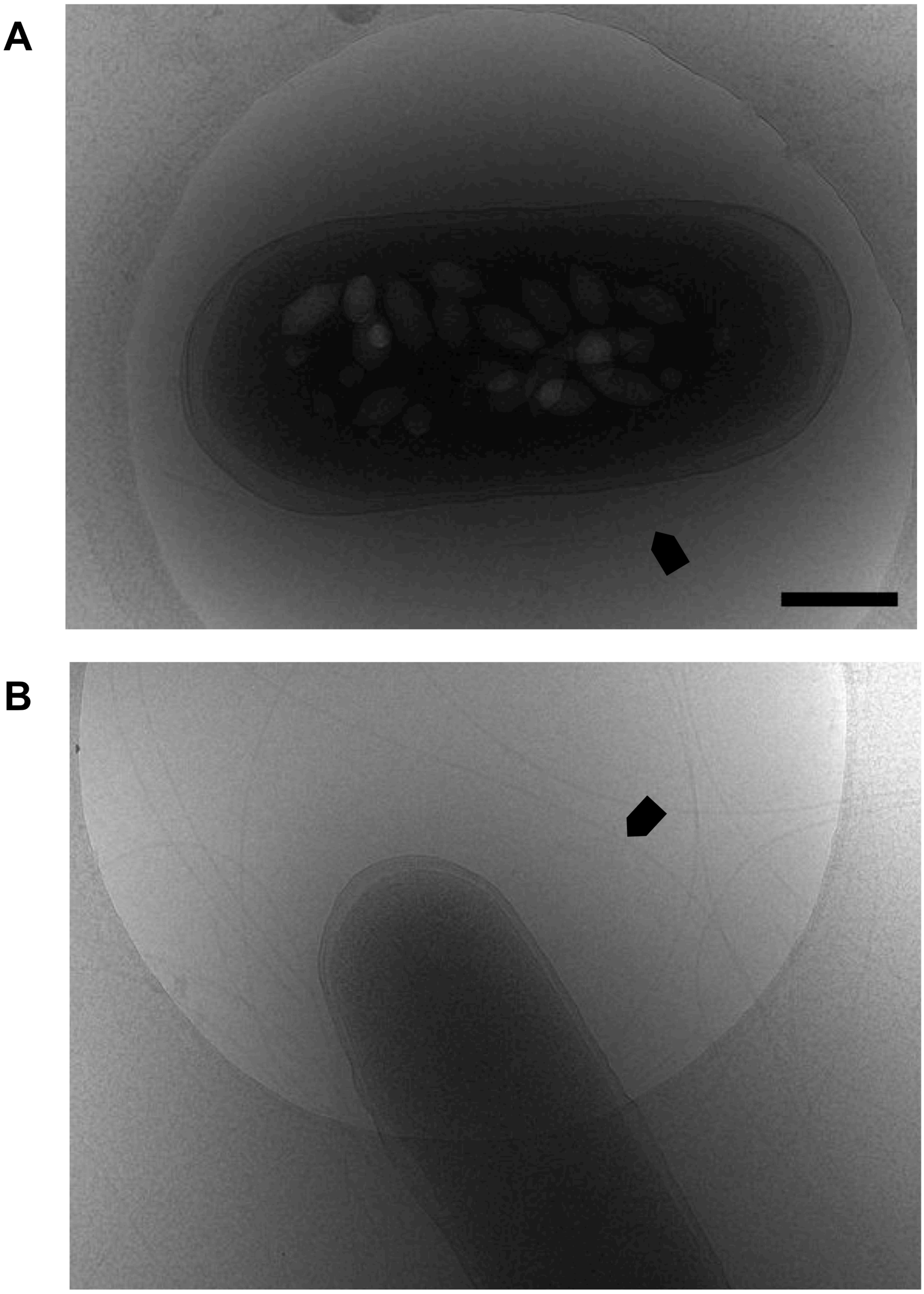

Figure S4. Cryo-Electron microscopy. Cell morphology of the WT (A) and *rsmC*$_{pro}$ (B) strains. Visible flagella are labeled by black arrowheads. Scale bar 200 nm.

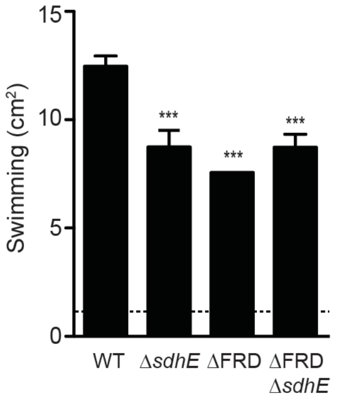

Figure S5. Both FRD and SdhE are required for maximal swimming. Swimming assay on WT, Δ*sdhE*, ΔFRD (Δ*frdABCD*::Cm), and Δ*sdhE*, ΔFRD (Δ*sdhE*, Δ*frdABCD*::Cm) mutants. Data shown are the means ± SD (*n*=3). The dashed line represents the limit of detection.

SUPPLEMENTARY REFERENCES

de Lorenzo, V., Herrero, M., Jakubzik, U. & Timmis, K. N. (1990). Mini-Tn5 transposon derivatives for insertion mutagenesis, promoter probing, and chromosomal insertion of cloned DNA in gram-negative eubacteria. *J Bacteriol* **172**, 6568-6572.

Fineran, P. C., Slater, H., Everson, L., Hughes, K. & Salmond, G. P. (2005). Biosynthesis of tripyrrole and beta-lactam secondary metabolites in *Serratia*: integration of quorum sensing with multiple new regulatory components in the control of prodigiosin and carbapenem antibiotic production. *Mol Microbiol* **56**, 1495-1517.

Grinter, N. J. (1983). A broad host range cloning vector transposable to various replicons. *Gene* **21**, 133-143.

Gristwood, T., McNeil, M. B., Clulow, J. S., Salmond, G. P. & Fineran, P. C. (2011). PigS and PigP regulate prodigiosin biosynthesis in *Serratia* via differential control of divergent operons, which include predicted transporters of sulfur-containing molecules. *J Bacteriol* **193**, 1076-1085.

Guzman, L. M., Belin, D., Carson, M. J. & Beckwith, J. (1995). Tight regulation, modulation, and high-level expression by vectors containing the arabinose PBAD promoter. *J Bacteriol* **177**, 4121-4130.

Herrero, M., de Lorenzo, V. & Timmis, K. N. (1990). Transposon vectors containing non-antibiotic resistance selection markers for cloning and stable chromosomal insertion of foreign genes in gram-negative bacteria. *J Bacteriol* **172**, 6557-6567.

Kaniga, K., Delor, I. & Cornelis, G. R. (1991). A wide-host-range suicide vector for improving reverse genetics in Gram-negative bacteria: inactivation of the *blaA* gene of *Yersinia enterocolitica*. *Gene* **109**, 137-141.

McNeil, M. B., Clulow, J. S., Wilf, N. M., Salmond, G. P. & Fineran, P. C. (2012). SdhE is a conserved protein required for flavinylation of succinate dehydrogenase in bacteria. *J Biol Chem* **287**, 18418-18428.

McNeil, M. B., Hampton, H. G., Hards, K. J., Watson, B. N., Cook, G. M. & Fineran, P. C. (2014). The succinate dehydrogenase assembly factor, SdhE, is required for the flavinylation and activation of fumarate reductase in bacteria. *FEBS letters* **588**, 414-421.

Przybilski, R., Richter, C., Gristwood, T., Clulow, J. S., Vercoe, R. B. & Fineran, P. C. (2011). Csy4 is responsible for CRISPR RNA processing in *Pectobacterium atrosepticum*. *RNA Biology* **8**, 517-528.

Ramsay, J. P., Williamson, N. R., Spring, D. R. & Salmond, G. P. (2011). A quorum-sensing molecule acts as a morphogen controlling gas vesicle organelle biogenesis and adaptive flotation in an enterobacterium. *Proceedings of the National Academy of Sciences of the United States of America* **108**, 14932-14937.

Thomson, N. R., Crow, M. A., McGowan, S. J., Cox, A. & Salmond, G. P. (2000). Biosynthesis of carbapenem antibiotic and prodigiosin pigment in *Serratia* is under quorum sensing control. *Mol Microbiol* **36**, 539-556.

Williamson, N. R., Fineran, P. C., Ogawa, W., Woodley, L. R. & Salmond, G. P. (2008). Integrated regulation involving quorum sensing, a two-component system, a GGDEF/EAL domain protein and a post-transcriptional regulator controls swarming and RhlA-dependent surfactant biosynthesis in *Serratia*. *Environmental microbiology* **10**, 1202-1217.
